# Not All SLAPs Are Created Equal: A Comparison of Patients with Planned and Incidental SLAP Repair Procedures

**DOI:** 10.1155/2019/9013935

**Published:** 2019-10-31

**Authors:** Mark C. Dougherty, J. Erik Kulenkamp, Haroutioun Boyajian, Jason L. Koh, Michael J. Lee, Lewis L. Shi

**Affiliations:** ^1^Resident, University of Iowa Hospitals and Clinics, Department of Neurosurgery, Iowa City, IA, USA; ^2^Resident, University of Minnesota, Department of Ophthalmology and Visual Neurosciences, Minneapolis, MN, USA; ^3^Resident, Henry Ford Health System, Department of Surgery, Detroit, MI, USA; ^4^Clinical Professor and Chairman, NorthShore University Health System, Department of Orthopaedic Surgery, Evanston, IL, USA; ^5^Director, NorthShore Orthopaedic Institute, Evanston, IL, USA; ^6^Associate Professor, University of Chicago Medicine, Department of Orthopaedic Surgery and Rehabilitation Medicine, Chicago, IL, USA

## Abstract

**Background:**

Epidemiological studies have shown a progressive increase in the rate of superior labrum anterior-posterior (SLAP) repair surgery after the year 2000. However, it is not clear whether this is due to increased recognition of isolated SLAP tears or increased SLAP repair performed secondarily during arthroscopy for other purposes.

**Hypothesis/Purpose:**

We hypothesized that both isolated SLAP repair and secondary SLAP repair increased with time and that patient age influenced the pathway to SLAP diagnosis and surgery—such that younger patients were more likely to have isolated SLAP repair surgery after being diagnosed in clinic.

**Study Design:**

Descriptive epidemiology study.

**Methods:**

Data were obtained from the MarketScan database from 2003 to 2013. CPT and ICD-9 codes were used to identify SLAP surgery patients and concomitant procedures. The timing of SLAP diagnosis relative to surgery was used to determine whether the injury was recognized preoperatively.

**Results:**

64,497 SLAP surgery patients were included. Preoperative SLAP diagnosis increased from 17.1% in 2003 to 44.6% in 2013. Patients diagnosed preoperatively were younger and had fewer concomitant procedures. Increasing age and concomitant rotator cuff tear (RCT) repair corresponded to lower odds of preoperative SLAP diagnosis.

**Discussion:**

Younger patients were more likely to have their SLAP tear diagnosed prior to surgery. Those diagnosed before surgery had fewer simultaneous procedures during their operations, suggesting that SLAP repair was more likely the primary operation. From 2003 to 2013, SLAP tears were increasingly recognized in the preoperative setting.

## 1. Introduction

Superior labrum anterior-to-posterior (SLAP) tears can lead to shoulder pain and instability in young and old patients [1]. SLAP diagnosis can be difficult in the office setting due to nonspecific clinical presentations and a high incidence of co-occurrence with other shoulder injuries; as a result, confirmation of the diagnosis often requires arthroscopy [2–9]. There is currently no standardized guideline for the surgical treatment of SLAP tears, but it is generally thought that young patients—especially athletes—should undergo SLAP repair, while older patients benefit more from biceps tenodesis [10–15].

Several large-scale epidemiological studies have identified a dramatic, unexplained increase in the number of SLAP repair surgeries after the year 2000 [16–18]. Work by our group corroborates those findings; we have also found that after 2009, the increased frequency of surgery for SLAP tears was mostly due to increasing rates of biceps tenodesis. It is unlikely that the true incidence of injury has changed with time; rather, the increased rate of SLAP repair is probably due to improved recognition of the injury. Studies have also suggested that many SLAP surgeries over this time period were performed on older patients [16, 18]. Given our knowledge of poorer outcomes for SLAP repair in older patients, we were inclined to further investigate the connection between age and SLAP repair. We wondered whether surgeons who identified and repaired SLAP tears during arthroscopy for another indication could account for a significant portion of the SLAP repair increase—especially among older patients. Perhaps SLAPs are easily missed when they are coincident with other shoulder injuries, as the clinical features of other injuries might mask the SLAP tear.

We therefore hypothesized that there would be several significant differences between SLAP surgery patients who were diagnosed with SLAP preoperatively and those who were not. Specifically, we hypothesized that patients with preoperative SLAP diagnoses were on average younger and had fewer concomitant shoulder procedures at the time of their SLAP repair than patients diagnosed with SLAP tears intraoperatively. With these differences, we aimed to shed light on the evolving epidemiology of SLAP surgery. To the best of our knowledge, no prior study has attempted to differentiate between these two groups of patients. We utilized data from a national database in order to ensure that our findings would be as generalizable as possible.

We also secondarily analyzed the subset of patients who underwent simultaneous rotator cuff tear (RCT) and SLAP repair. We specifically chose RCT repair for further evaluation because rotator cuff tears are easily recognized prior to surgery and have a substantial clinical impact; thus, RCT repair may be one of the more common procedures during which a surgeon might intraoperatively identify and repair a SLAP tear. RCT repairs are also frequently done in older patients, so that situation may contribute to the high rate of SLAP repairs seen in older patients [19]. Given the relative ease of identifying a torn rotator cuff and the high annual rate of RCT repair (substantially higher than SLAP repair), it is likely that RCT repair was the primary indication for surgery in most cases when RCT and SLAP repairs occurred simultaneously. Thus, we hypothesized that patients with concomitant SLAP and RCT repair surgeries were older and less often diagnosed with SLAP preoperatively than those without concomitant RCT repair.

## 2. Methods

Patient data in this study were obtained from the Truven Health MarketScan Commercial Database with Medicare Supplemental (http://marketscan.truvenhealth.com/; Ann Arbor, MI, USA), a fee-for-service private insurance claims database with approximately 55 million unique patients in the United States from 2003 to 2013. The database contains information on diagnoses, procedures, patient demographic data, and payments made for a given procedure. Current Procedure Technology (CPT) and International Classification of Diseases-Ninth Revision, Clinical Modification (ICD-9) codes were used to identify relevant procedures and diagnoses, respectively. We identified patients in the database who had exactly one arthroscopic SLAP repair surgery coded (CPT code 29807) between 2003 and 2013. Among these patients, we identified those who also had an RCT repair procedure (CPT codes 29827, 23410, 23412, and 23420) coded on the same day as their SLAP repair. Patients with less than 6 uninterrupted months of claims data before and after SLAP surgery were excluded to minimize the risk of missing key diagnosis and procedure codes. The timing of a patient's earliest SLAP diagnosis code (ICD-9 code 840.7) was used to determine whether the SLAP lesion was recognized prior to SLAP surgery. CPT codes were also used to characterize other concomitant shoulder surgeries (e.g., 29826 subacromial decompression).

Two-sample *t*-tests were used to compare the mean ages and mean number of concomitant shoulder procedures of the preoperative and intraoperative SLAP diagnosis groups. A two-sample *t*-test was also used to compare the mean ages of the RCT repair and non-RCT repair groups. Multivariate logistic regression analysis was used to evaluate the odds of preoperative diagnosis based on age and concomitant RCT repair. One-way analysis of variance (ANOVA) with post hoc Scheffe pairwise testing was used to determine the significance of differences between specific age groups (e.g., 10–19, 20–29). Given our large sample size, we used *p* < 0.001 as the cutoff for statistical significance for all calculations. All statistical analyses were performed using Stata 14.0 (College Station, TX).

## 3. Results

**Figure 1:**
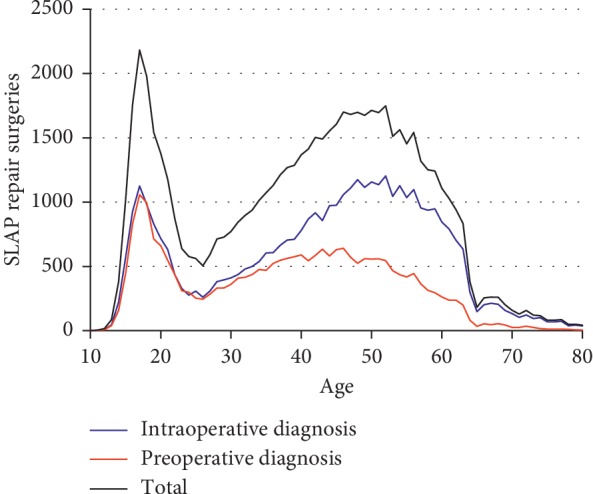
Total number of SLAP repair procedures performed 2003–2013 by patient age, with preoperative diagnosis (red), without preoperative diagnosis (blue), and total (black). The peaks of a biphasic distribution can be seen at ages 17 and 52.

A total of 64,497 SLAP surgery patients were included in our study; of those, 24,438 were diagnosed with a SLAP lesion prior to SLAP surgery, and 40,059 were not. Patients who were diagnosed preoperatively were significantly younger than those who were not (37.6 vs. 43.2 years; *p* < 0.0001). Likewise, logistic regression demonstrated that increasing age (OR 0.982 per year, *p* < 0.001) and concomitant RCT repair (OR 0.594, *p* < 0.001) corresponded to lower odds of preoperative SLAP diagnosis.  Figure 1 illustrates the relative proportions of SLAP repair patients who were diagnosed prior to surgery based on the patient's age. The total number of SLAP surgeries based on patient age had a biphasic distribution, with one peak at age 17 years and another from ages 46 to 52 years (Figure 1).

**Figure 2:**
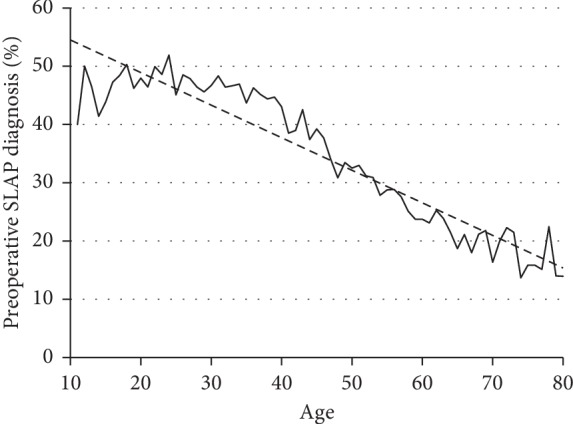
Percentage of patients with SLAP diagnosis prior to surgery. Dashed line represents line of best fit.

Nearly half of the patients under 30 were diagnosed preoperatively, whereas a substantially smaller percent of older patients was diagnosed preoperatively. Increasing age corresponded to a decreasing percentage of patients whose SLAP was diagnosed prior to surgery (Figure 2). 87.5% of the patients had one or more concomitant shoulder procedures performed at the time of SLAP repair, the most common of which were subacromial decompression (CPT 29826; 56.0% of patients) and capsulorrhaphy (CPT 29806; 26.6% of patients). Patients who were diagnosed with SLAP tear preoperatively had significantly fewer concomitant surgical procedures (2.67 ± 1.26) than those who were not (3.04 ± 1.28; *p* < 0.0001). Similarly, younger patients had fewer concomitant procedures on average than older patients; for example, patients 10–19 years old averaged 2.07 ± 0.90 concomitant shoulder procedures (including SLAP repair), compared to 3.42 ± 1.26 in the 50–59 year age range—a significant difference (*p* < 0.001).

Of the 64,497 SLAP repair patients in our cohort, 17,563 (27.2%) had concomitant RCT repair. However, only 18.2% of the patients with preoperative SLAP diagnosis had concomitant RCT repair compared to 32.7% of the patients without preoperative SLAP diagnosis. Independent of SLAP diagnosis status, patients with concomitant RCT repair were significantly older than non-RCT repair patients (51.6 vs. 37.1 years; *p* < 0.0001) and had more concomitant procedures than non-RCT patients, even without counting the RCT repair (2.00 vs. 1.49, *p* < 0.0001).

**Figure 3:**
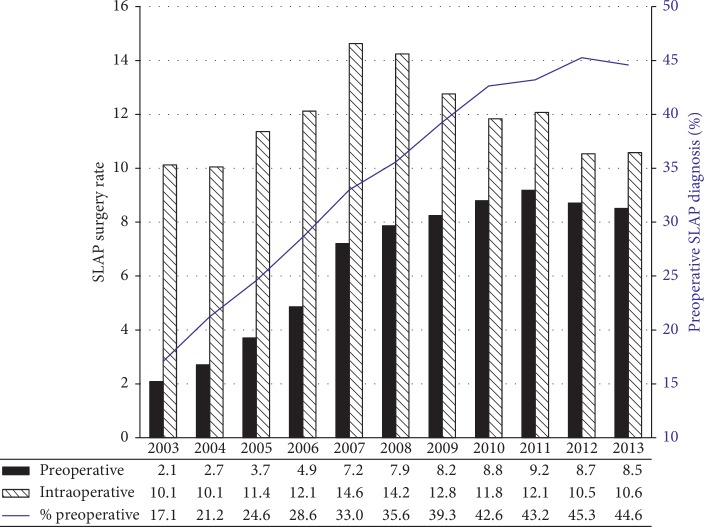
Left axis: SLAP repairs per 100,000 patients; ^*∗*^ with (black) or without (striped) preoperative SLAP diagnosis. Right axis (blue): percent of SLAP repairs with SLAP diagnosis prior to surgery. ^*∗*^2003 and 2013 included only 6 months of data, so numbers from these years were doubled for accurate comparison.

As suggested by other studies, we demonstrated that the overall rate of SLAP repair increased over much of the first decade of the new millennium, peaking in 2008 (Figure 3). There was a slight decrease from 2008 to 2013, but rates remained well above 2003 levels. The increase over time was due to increased rates of repair of both diagnosis groups—those with SLAP lesions diagnosed preoperatively and those diagnosed intraoperatively—but the relative contributions of each group changed with time. From 2003 to 2007, the rate of repair in both groups increased substantially. In contrast, from 2008 to 2013, SLAP repairs following intraoperative diagnosis declined, whereas those following preoperative diagnosis remained fairly constant. The percentage of SLAP repair patients that were diagnosed before surgery increased consistently over time, from a low of 17.1% in 2003 to a peak of 45.3% in 2012 (Figure 3).

## 4. Discussion

While increases in the incidence and repair of SLAP lesions have been noted in the literature, no previously published work has evaluated the impact that age has on how a SLAP tear is diagnosed in those who eventually undergo SLAP repair surgery [16–18, 20]. Our results confirm our suspicion that younger patients were significantly more likely to have their SLAP tear diagnosed prior to surgery. Those who were diagnosed before surgery had significantly fewer concomitant procedures, suggesting that SLAP repair was more likely the primary operation. Ultimately, SLAP repair occurring as a secondary, unplanned procedure may partly explain the high rate of SLAP repair in older patients. Our example of concomitant RCT repair supports this conclusion: RCT repair patients were older, less likely to have a recognized SLAP tear prior to surgery, and had more concomitant procedures than non-RCT repair patients. In other words, these patients were older and had more complicated shoulder pathology than their counterparts. On the one hand, this is a somewhat obvious finding, given that we are comparing patients with one specific procedure (SLAP repair) to others with two specific procedures (SLAP repair and RCT repair). On the other hand, it is easy to imagine why such patients may not have SLAP tears recognized prior to surgery—the more complicated the pathology, the more difficult it is to identify features specific to SLAP tears, which might explain the lower rate of SLAP diagnosis prior to arthroscopy. The corollary is also true: in younger patients, who tend to have less complicated shoulder pathology, SLAP tears are more easily identified without arthroscopy. To confirm these suspicions, further investigation is necessary; this study only evaluated patients who underwent SLAP repair, so we do not have any insight into intraoperative SLAP tear diagnoses that were not subsequently repaired, including those addressed via biceps tenodesis. Furthermore, insurance claims do not contain information on specific symptomatology, so we cannot determine whether SLAP tears identified during surgery were actually symptomatic during preoperative evaluation.

Intraoperative SLAP diagnosis does not fully explain the changing rate of SLAP repair over time because the percentage of SLAP surgeries that had a preoperative SLAP diagnosis actually increased over the period studied. This suggests that, from 2003 to 2013, the orthopaedic community became better at identifying SLAP tears in the preoperative setting. Whether this recognition was due to higher clinical suspicion in the office, better physical examination, or improved imaging technology is unclear. Notably, there was a temporary increase in intraoperatively diagnosed SLAP repairs in the middle portion of our study period, peaking in 2007 and 2008, but this increase was essentially gone by 2012 (Figure 3). Although somewhat speculative, our best explanation for this observation is that the orthopaedic community began to identify, and then repair, more and more SLAP lesions before collectively realizing that many SLAP tears should not be repaired. Further investigation showing the rates of SLAP diagnosis independent of SLAP surgery is needed to support this hypothesis.

The MarketScan database allows for a unique analysis of the procedures in question for a variety of reasons. Its national scale reduces the impact of normal variations in physician practices. Its size enables us to identify small differences that smaller datasets cannot. Few studies have utilized a database of this magnitude to evaluate rates of concomitant surgery, and fewer still have stratified specifically by patient age [21–24]. We believe that our findings will therefore help advance our understanding of how patient characteristics affect SLAP repair. In the absence of properly conducted randomized controlled trials, indirectly derived data, such as this, are the best information available for guiding treatment.

## 5. Limitations

This analysis depends on accurate and consistent billing codification by physicians. We cannot distinguish between true clinical differences and physician-to-physician coding variations. For example, some physicians may enter nonspecific codes such as 719.41 (shoulder pain) until a SLAP lesion is arthroscopically confirmed. Furthermore, surgeons may not list every unique diagnosis while billing—especially in patients with several diagnoses—as they are likely paid the same amount for an office visit regardless of the diagnosis listed; in contrast, we suspect surgeons more often billed for every surgical procedure performed, as doing so probably impacts their payment. Thus, our method of analysis is more likely to miss a suspected diagnosis than a surgical procedure, so we may underestimate the amount of SLAP tears that were suspected prior to surgery. Stated differently, we may overestimate the prevalence of incidental SLAP repairs (SLAPs diagnosed intraoperatively). However, these coding ambiguities are unlikely to vary by age or year, so the differences we found between young and old patients and changes over time are still noteworthy.

Insurance database analyses also lack information on clinical outcome measures, such as pain scores and postoperative functional recovery. Therefore, we cannot directly compare outcome measures between groups, such as old versus young patients, or patients with and without concomitant rotator cuff repair. Finally, although surgeon experience is an obvious and important factor in clinical decision-making, the MarketScan database does not identify individual providers or institutions from which each billing code came. Hence, we cannot comment on whether surgeon experience, institutional volume, private versus academic setting, or various other provider-related factors impact our findings. Although in some ways this is a limitation to our study, it also makes our findings more generalizable since we encompass all practice settings and all ranges of orthopaedic surgeons within the United States.

## 6. Conclusions

Younger patients were more likely to have their SLAP tear diagnosed prior to surgery. Those who were diagnosed before surgery had fewer operations, suggesting that SLAP repair was more likely the primary operation. From 2003 to 2013, SLAP tears were increasingly recognized in the preoperative setting.

## Data Availability

The data used to support the findings of this study were supplied by the Truven MarketScan database (https://marketscan.truvenhealth.com/marketscanportal/) under license and so cannot be made freely available. Requests for access to these data should be made to Truven Health (now a subsidiary of IBM) at scanmar@us.ibm.com. For more information about MarketScan, please see http://truvenhealth.com/Your-Healthcare-Focus/Government/Analytic-Research/MarketScan.

## References

[1] Powell S. E., Nord K. D., Ryu R. K. N. (2012). The diagnosis, classification, and treatment of SLAP lesions. *Operative Techniques in Sports Medicine*.

[2] Aydin N., Sirin E., Arya A. (2014). Superior labrum anterior to posterior lesions of the shoulder: diagnosis and arthroscopic management. *World Journal of Orthopedics*.

[3] Chandnani V. P., Yeager T. D., DeBerardino T. (1993). Glenoid labral tears: prospective evaluation with MRI imaging, MR arthrography, and CT arthrography. *American Journal of Roentgenology*.

[4] Jee W.-H., McCauley T. R., Katz L. D., Matheny J. M., Ruwe P. A., Daigneault J. P. (2001). Superior labral anterior posterior (SLAP) lesions of the glenoid labrum: reliability and accuracy of MR arthrography for diagnosis. *Radiology*.

[5] Kibler W. B., Sciascia A. (2015). Current practice for the diagnosis of a SLAP lesion: systematic review and physician survey. *Arthroscopy: Journal of Arthroscopic & Related Surgery*.

[6] Magee T. (2015). Usefulness of unenhanced MRI and MR arthrography of the shoulder in detection of unstable labral tears. *American Journal of Roentgenology*.

[7] O’Kane J. W., Toresdahl B. G. (2014). The evidenced-based shoulder evaluation. *Current Sports Medicine Reports*.

[8] Popp D., Schöffl V. (2015). Superior labral anterior posterior lesions of the shoulder: current diagnostic and therapeutic standards. *World Journal of Orthopedics*.

[9] Sheridan K., Kreulen C., Kim S., Mak W., Lewis K., Marder R. (2015). Accuracy of magnetic resonance imaging to diagnose superior labrum anterior-posterior tears. *Knee Surgery, Sports Traumatology, Arthroscopy*.

[10] Brockmeyer M., Tompkins M., Kohn D. M., Lorbach O. (2016). SLAP lesions: a treatment algorithm. *Knee Surgery, Sports Traumatology, Arthroscopy*.

[11] Kibler W. B., Sciascia A. (2016). Current practice for the surgical treatment of SLAP lesions: a systematic review. *Arthroscopy: Journal of Arthroscopic & Related Surgery*.

[12] McCormick F., Bhatia S., Chalmers P., Gupta A., Verma N., Romeo A. A. (2014). The management of type II superior labral anterior to posterior injuries. *Orthopedic Clinics of North America*.

[13] Neuman B. J., Boisvert C. B., Reiter B., Lawson K., Ciccotti M. G., Cohen S. B. (2011). Results of arthroscopic repair of type II superior labral anterior posterior lesions in overhead athletes. *American Journal of Sports Medicine*.

[14] Provencher M. T., McCormick F., Dewing C., McIntire S., Solomon D. (2013). A prospective analysis of 179 type 2 superior labrum anterior and posterior repairs. *American Journal of Sports Medicine*.

[15] Erickson J., Lavery K., Monica J., Gatt C., Dhawan A. (2015). Surgical treatment of symptomatic superior labrum anterior-posterior tears in patients older than 40 years. *American Journal of Sports Medicine*.

[16] Zhang A. L., Kreulen C., Ngo S. S., Hame S. L., Hame J. C., Gamradt S. C. (2012). Demographic trends in arthroscopic SLAP repair in the United States. *American Journal of Sports Medicine*.

[17] Onyekwelu I., Khatib O., Zuckerman J. D., Rokito A. S., Kwon Y. W. (2012). The rising incidence of arthroscopic superior labrum anterior and posterior (SLAP) repairs. *Journal of Shoulder and Elbow Surgery*.

[18] Ablove R. H., Allison A., Baer G. (2014). The incidence and demographics of shoulder repair in Wisconsin, 2002-2010. *WMJ: Official Publication of the State Medical Society of Wisconsin*.

[19] Jain N. B., Higgins L. D., Losina E., Collins J., Blazar P. E., Katz J. N. (2014). Epidemiology of musculoskeletal upper extremity ambulatory surgery in the United States. *BMC Musculoskeletal Disorders*.

[20] Vogel L. A., Moen T. C., Macaulay A. A. (2014). Superior labrum anterior-to-posterior repair incidence: a longitudinal investigation of community and academic databases. *Journal of Shoulder and Elbow Surgery*.

[21] Cancienne J. M., Brockmeier S. F., Werner B. C. (2016). Tobacco use is associated with increased rates of infection and revision surgery after primary superior labrum anterior and posterior repair. *Journal of Shoulder and Elbow Surgery*.

[22] Shields E., Thirukumaran C., Thorsness R., Noyes K., Voloshin I. (2015). An analysis of adult patient risk factors and complications within 30 days after arthroscopic shoulder surgery. *Arthroscopy: Journal of Arthroscopic & Related Surgery*.

[23] Martin C. T., Gao Y., Pugely A. J., Wolf B. R. (2013). 30-day morbidity and mortality after elective shoulder arthroscopy: a review of 9410 cases. *Journal of Shoulder and Elbow Surgery*.

[24] Yeranosian M. G., Arshi A., Terrell R. D., Wang J. C., McAllister D. R., Petrigliano F. A. (2014). Incidence of acute postoperative infections requiring reoperation after arthroscopic shoulder surgery. *American Journal of Sports Medicine*.

